# Deep Learning Based on ACR TI-RADS Can Improve the Differential Diagnosis of Thyroid Nodules

**DOI:** 10.3389/fonc.2021.575166

**Published:** 2021-04-27

**Authors:** Ge-Ge Wu, Wen-Zhi Lv, Rui Yin, Jian-Wei Xu, Yu-Jing Yan, Rui-Xue Chen, Jia-Yu Wang, Bo Zhang, Xin-Wu Cui, Christoph F. Dietrich

**Affiliations:** ^1^ Sino-German Tongji-Caritas Research Center of Ultrasound in Medicine, Department of Medical Ultrasound, Tongji Hospital, Tongji Medical College, Huazhong University of Science and Technology, Wuhan, China; ^2^ Department of Artificial Intelligence, Julei Technology Company, Wuhan, China; ^3^ Department of Ultrasound, Affiliated Renhe Hospital of China Three Gorges University, Yichang, China; ^4^ Department of Ultrasound, The First Affiliated Hospital of Zhengzhou University, Zhengzhou, China; ^5^ Department of Ultrasound, Wuchang Hospital, Wuhan University of Science and Technology, Wuhan, China; ^6^ Department of Ultrasonic Imaging, Xiangya Hospital, Central South University, Changsha, China; ^7^ Department of General Internal Medicine, Kliniken Hirslanden Beau-Site, Bern, Switzerland

**Keywords:** artificial intelligence, thyroid imaging reporting and data system (TI-RADS), ultrasound, thyroid cancer, deep learning

## Abstract

**Objective:**

The purpose of this study was to improve the differentiation between malignant and benign thyroid nodules using deep learning (DL) in category 4 and 5 based on the Thyroid Imaging Reporting and Data System (TI-RADS, TR) from the American College of Radiology (ACR).

**Design and Methods:**

From June 2, 2017 to April 23, 2019, 2082 thyroid ultrasound images from 1396 consecutive patients with confirmed pathology were retrospectively collected, of which 1289 nodules were category 4 (TR4) and 793 nodules were category 5 (TR5). Ninety percent of the B-mode ultrasound images were applied for training and validation, and the residual 10% and an independent external dataset for testing purpose by three different deep learning algorithms.

**Results:**

In the independent test set, the DL algorithm of best performance got an AUC of 0.904, 0.845, 0.829 in TR4, TR5, and TR4&5, respectively. The sensitivity and specificity of the optimal model was 0.829, 0.831 on TR4, 0.846, 0.778 on TR5, 0.790, 0.779 on TR4&5, versus the radiologists of 0.686 (*P*=0.108), 0.766 (*P*=0.101), 0.677 (*P*=0.211), 0.750 (*P*=0.128), and 0.680 (*P*=0.023), 0.761 (*P*=0.530), respectively.

**Conclusions:**

The study demonstrated that DL could improve the differentiation of malignant from benign thyroid nodules and had significant potential for clinical application on TR4 and TR5.

## Introduction

With the utilization of high-frequency ultrasound in clinical practice and the gradual enhancement of public health awareness especially on physical examination, the detection of thyroid nodules (TN) has increased, with a prevalence ranging from 19% to 68% in the general unselected population (1, 2). Moreover, the incidence rate of thyroid cancer has continued to increase and is now the highest cause of cancer in women under 30 years old in China (3, 4). Ultrasound has an irreplaceable role in early detection of thyroid cancer due to its accessibility, high resolution, safety, using no radiation, and provision of real-time imaging with multi-dimensions. Experience and skills of different operators influence the accurate differential diagnosis of TN, and thus, a precise and independent method is needed.

To implement standardized management of the thyroid nodules, the Thyroid Imaging Reporting and Data System (TI-RADS) Committee of American College of Radiology (ACR) published a white paper in 2017 that presented a new risk stratification system from TR1 to TR5 for classifying thyroid nodules by adding scores of the five characteristics on ultrasound, composition, echogenicity, shape, margin, and echogenic foci (5). Recommendations for biopsy or ultrasound follow-up are determined on the nodule’s ACR TI-RADS categories and its maximum diameter (6), which provides clarity for the further diagnosis and treatment measures. The guidance of ACR TI-RADS has been proven to be a reliable tool to assist doctors to differentiate between malignant and benign thyroid nodules (7–11), with a pooled sensitivity of 0.79 (95% confidence interval [CI] = 0.77-0.81) and a pooled specificity of 0.71 (95% CI = 0.70-0.72) (12, 13).

Artificial Intelligence (AI) is of unique value for its time-saving and non-dependence on radiologist’s experience, and performs extremely well on the tasks of detection, extraction and classification of the TN on ultrasound images (14–18). Recently, AI has accomplished many complex tasks on thyroid ultrasound, such as the differentiation of malignant from benign thyroid nodules using ultrasound images from multiple cohorts (19), developing a deep learning (DL) algorithm to decide whether a TN should undergo a biopsy (16), using ultrasound elastography to improve thyroid nodule discrimination (20) and applying ultrasound images to predict metastasis in the cervical lymph nodes (21, 22).

However, there are still some flaws in these studies. First, pathological results of some nodules are missing in almost all of the published studies (19). Second, all types of thyroid nodules were included, but some nodules are easily diagnosed by doctors and AI is not that necessary. For example, cystic nodules are usually echoless with clear boundaries and it is not surprising that AI performs diagnosing them as benign.

ACR TI-RADS is popularly used in routine clinical practice, and has proven value. It is still an open question if the combination of DL and TI-RADS can improve the differential diagnosis of TNs. TR1, TR2, TR3 have a very low (less than 5%) chance of malignancy (6) and the necessity for them to proceed AI analysis seem less sufficient. Adversely, malignant thyroid nodules were most distributed in TR4 and TR5. However, it is difficult for radiologists to differentiate benign from malignant nodules in the same category causing that they have same ultrasound descriptive features (23). A non-invasive method such as DL is needed to avoid the need for unnecessary biopsy.

The purpose of this study was to evaluate whether DL based on ACR TI-RADS category 4 and 5 could improve the differentiation of malignant from benign thyroid nodules, and explore the clinical application potential for it.

## Materials and Methods

### Source of the Data

This study was approved by the Ethics Committee of Tongji Medical College of Huazhong University of Science and Technology. Informed consent from the patients was exempted (2019S1233). All ultrasound images included were consecutively acquired from 11 operators with more than 5 years of experience from Tongji hospital, Wuhan, China (internal cohort), and Xiangya Hospital of Central South University, Changsha, China (external cohort) from June 2017 to April 2019. Ultrasound equipment manufactured by GE Healthcare (LOGIQ E9, LOGIQ S7), Samsung (RS80A), and Philips (EPIQ5, EPIQ7 and IU22), was used to generate the thyroid ultrasound images. Ultrasound images were derived from the picture archiving and communication system (PACS) workstations.

### Images Enrolments and Grouping

The inclusion criteria for thyroid nodules in this study were patients who 1) underwent total or nearly total thyroidectomy or lobectomy; 2) had pathological specimens examined within one month after US examination; 3) had complete medical information including preoperative ultrasound of the thyroid nodules; 4) had no previous surgical treatment or FNA performed on the nodules.

Exclusion criteria were lesions 1) with unsatisfactory ultrasound image quality; 2) where the finding on ultrasound did not match with the pathological results in position or size; 3) received chemotherapy and/or radiotherapy such as iodine 131 treatment before ultrasound examination.

From June 2nd, 2017 to April 23th, 2019, 4910 thyroid images from 2779 consecutive patients and 213 thyroid images from 195 consecutive patients with confirmed postoperative pathological results were retrospectively collected in Tongji hospital and Xiangya Hospital of Central South University. Three doctors (C.R, Y.R, and W.G) scored these images on the five features according to ACR TI-RADS lexicon (6). The opinion of the third was referred to for cases where the first opinions differed. Only nodules of TI-RADS category 4 (dataset I) and category 5 (dataset II) were enrolled, and they were merged together as new dataset III (i.e. combination of ACR TI-RADS 4 and 5). In accordance with the pathological results, images of each category were sorted out into a benign group and a malignant group.

### Establishment of Training Set and Test Set

**Figure 1:**
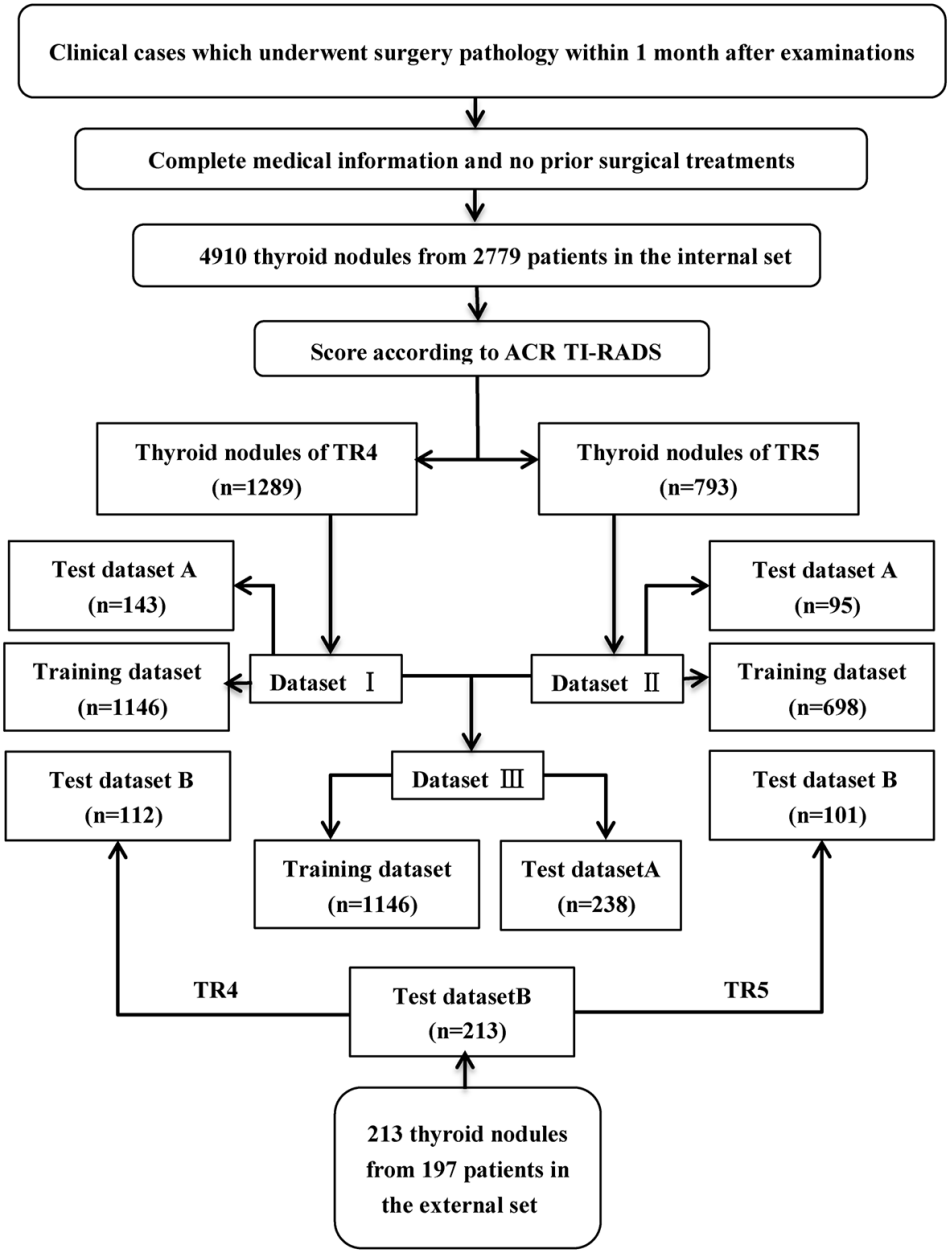
Workflow of the construction of the training and test dataset.

Each inner dataset (I, II, III) was randomly divided into two sets, 90% for training and validation, and the residual 10% (test set A) for testing. In addition, another independent outer test set (test set B) was obtained for testing as well. Three convolutional neutral Network (CNN) models named ResNet-50, Inception-Resnet v2, Desnet-121 were used for analysis. The workflow of the selection and construction is shown in **Figure 1**.

Three independent experienced radiologists (X.J and Y.Y and Z.B) with 8 years, 9 years and 24 years of experience, respectively, read the images and gave their judgments according to the ACR TI-RADS lexicon (5, 6) and their own clinical experience. If their opinions did not agree, the opinion of the most senior radiologist was used.

### Processing of Ultrasound Images

Nodules were manually marked, and the region of interest (ROI) of the thyroid nodules was cut out using rectangular boxes by Image J (version 1.48, National Institutes of Health, USA) by a radiologist, in which the cropped images include the entire thyroid nodule. All the images were resized to 299 × 299 pixels to standardize the distance scale. Due to the limited quantity of the dataset, augmentation strategy was introduced to process the images. All preprocessing steps were conducted using the Keras Image Data Generator and then fed into the input.

### Construction of CNNs

The tasks on three sets (datasets I, II, and III) were trained on three pre-trained convolutional neural networks, named ResNet50, Inception-ResNet v2, Desnet 121, respectively. The initialization set of the parameters of these models was referred to ImageNet and obtained from Keras Team (https://github.com/keras-team/keras-applications/releases). The learning rate was set to 0.03 and decelerated by a factor of 0.1 for each 50 epochs when the accuracy had no further improvement in the training and validation set. Model learning continued until the least loss of the validation set appeared and the final model was determined accordingly. Optimizer of Stochastic Gradient Descent (SGD) and binary cross entropy technique were used to decrease loss in the process in CNNs. All models were trained in Python 3.6.2 (https://www.python.org) by using a computer with a GeForce GTX 2080 Ti graphics processing unit (NVIDIA, Santa Clara, California, America), a Core i9-9900K central processing unit (Intel, Santa Clara, California, America).

**Figure 3:**
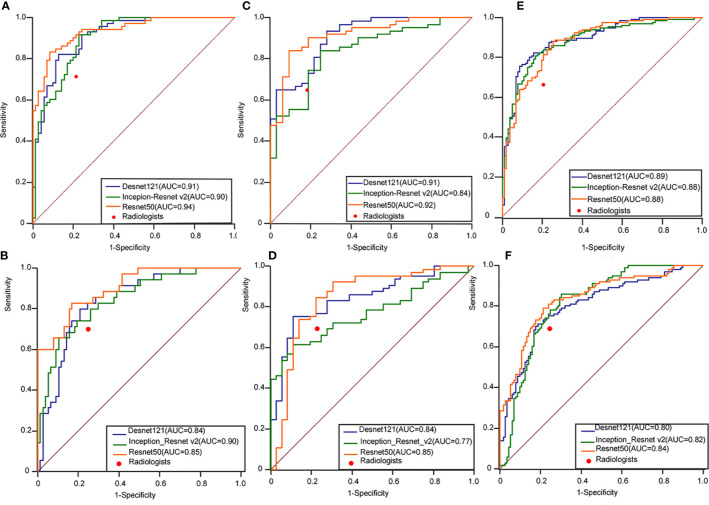
Performance of the ensemble D-CNN models in identifying patients with thyroid cancer in TR4 **(A)**, TR5 **(C)**, and TR4&5 **(E)** on three inner test datasets and TR4 **(B)**, TR5 **(D)**, and TR4&5 **(F)** on three outer test datasets. The red dots on each ROC curve demonstrate the performance of the radiologists. AUC, area under the curve; DCNN, deep convolutional neural network; ROC, receiver operating characteristics curve.

The class activation mapping (CAM) technique was also used to produce the heated maps which indicated the focus of the CNN model’s prediction (24, 25). The CAM can be regarded as the multiplication of the feature maps of the pooling layers and weight of the fully connected layer, which prevented loss of the special information when feature maps were transferred to eigenvector. It highlighted the specific discriminative regions demonstrated as thyroid cancer by CNN. Packages Matplotlib 3.1.1 (https://matplotlib.org) and Open cv-Python 3.4.4.19 (https://github.com/skvark/opencv-python) was employed to generate heatmaps (**Figure 3**).

### Statistical Analysis

The performance of the three algorithms was measured by the area under the receiver operating characteristic curve (AUROC) of the training and test dataset. The cut-off value was obtained as the threshold value when the Youden index reached its maximum. Then, the accuracy, sensitivity, specificity, positive predictive value (PPV), and negative predictive value (NPV) of each method were calculated to judge the performance of the experts and the CNNs. Delong test was introduced to evaluate the statistical difference between different AUCs. Ninety-five percent confidence interval (CI) was utilized to estimate the range of these evaluation values. P-value less than 0.05 with two tailed was considered statistically significant. Interobserver agreements on thyroid nodules were assessed using Kruskal–Wallis test. Kappa values were interpreted as follows. Less than 0.20 mean poor agreement, from 0.20 to 0.40 mean fair agreement, from 0.40 to 0.60 imply moderate agreement, between 0.60 and 0.80 imply substantial agreement, and excellent agreement tend to be over 0.80. F score was introduced to measure the efficiency of the CNNs while taking both Precision and Recall into account, the formula is as follows. When β = 1, the F1 score improves Precision and Recall as much as possible, and makes the difference between the two as small as possible.

$$F\;score=\left(1+\beta^{2}\right)\times \frac{Precision\times Recall}{\left(\beta^{2}\times Precision\right)+Recall}$$

The curve of ROC was performed and portraited using the pROC package of R software (version 1.8) and MedCalc (version 11.2, Ostend, Belgium). Outcome of evaluation values was also obtained by SPSS (version 22.0, IBM, Chicago) and R software.

## Results

### Characteristics of the Thyroid Nodules

A total of 2295 thyroid images from 1593 patients were used in this research (**Table 1**). In the internal cohort, the mean age of all patients was 45.48 ± 10.33, of which 1059 were woman, 337 were men. In the external cohort, the mean age of all patients was 45.54 ± 11.82, of which 150 were woman, 47 were men. 1146 thyroid images of TR4 and 698 thyroid images of TR5 were enrolled in training set in this research, which consisted of 637 benign images and 509 malignant images in the former, 297 benign images and 401 malignant images in the latter. 143 thyroid images of TR4 and 95 thyroid images of TR5 were predicted for the internal test in this research, while 112 of TR4 and 101 of TR5 for the external test. The characteristics of the thyroid nodules in five ACR TI-RADS features were summarized in **Table 2**.

**Table 1:** Basic information of the patients.

	Internal dataset (n=1396)	External dataset (n=197)
Age (year)	45.48 ± 10.33 (8-71)	45.54 ± 11.82 (16-77)
≤20	13(0.9)	1(0.5)
20-30	85(6.1)	27(13.7)
30-40	281(20.1)	37(18.8)
40-50	549(39.3)	62(31.5)
≥50	468(33.5)	70(35.5)
Gender		
Male	337(24.1)	47(23.9)
Female	1059(75.9)	150(76.1)

**Table 2:** Characteristics of the thyroid nodules in internal set enrolled in this survey.

	Task1			Task2			Task3		
	Training dataset(n=1146)	Test dataset A (n=143)	Test dataset B (n=112)	Training dataset(n=698)	Test dataset A(n=95)	Test dataset B (n=101)	Training dataset (n=1844)	Test dataset A (n=238)	Test dataset B (n=213)
Pathology									
benign	637(55.6)	70(49.0)	77(68.8)	297(42.6)	32(33.7)	36(35.6)	934(50.7)	102(42.9)	113(53.1)
malignant	509(44.4)	73(51.0)	35(31.2)	401(57.4)	63(66.3)	65(64.4)	910(49.3)	136(57.1)	100(46.9)
Diameter (mm)									
≤ 0.5	221(19.3)	26(18.1)	19(17.0)	93(13.3)	14(14.7)	9(8.9)	314(17.0)	40(16.8)	28(13.1)
0.5‐1.0	431(37.6)	57(39.9)	55(49.0)	295(42.3)	41(43.2)	35(34.7)	726(39.4)	98(41.2)	90(42.3)
1.0‐2.0	176(15.4)	39(27.3)	28(25.0)	125(17.9)	25(26.3)	29(28.7)	301(16.3)	64(26.9)	57(26.8)
> 2.0	318(27.7)	21(14.7)	10(9.0)	185(36.5)	15(15.8)	28(27.7)	503(23.3)	36(15.1)	38(17.8)
Internal Composition									
Cystic/partially cystic/spongifom	0(0)	0(0)	0(0)	0(0)	0(0)	0(0)	0(0)	0(0)	0(0)
Mixed	48(4.2)	7(4.9)	6(5.3)	3(0.4)	1(1.1)	1(1.0)	51(2.8)	8(3.4)	7(3.3)
Solid/almost solid	1098(95.8)	136(95.1)	106(94.6)	695(99.6)	94(98.9)	100(99.0)	1793(97.2)	230(96.6)	206(96.7)
Echogenicity									
Anechoic	0(0)	0(0)	0(0)	0(0)	0(0)	0(0)	0(0)	0(0)	0(0)
Hyperechoic/ isoechoic	30(2.6)	6(4.2)	4(3.6)	5(0.7)	1(1.1)	0(45)	35(1.9)	7(2.9)	4(1.9)
Hypoechoic	1113(97.1)	137(95.8)	107(95.5)	681(95.6)	92(96.8)	100(99.0)	1814(98.3)	229(96.2)	207(97.2)
Very hypoechoic	3(0.3)	0(0)	1(0.9)	12(1.7)	2(2.1)	1(1.0)	15(0.8)	2(0.8)	2(0.9)
Shape									
Wider-than-tall	1143(99.7)	142(99.3)	112(100.0)	478(68.5)	65(68.4)	70(69.3)	1621(87.9)	207(87.0)	182(85.4)
Taller-than-wide	3(0.3)	1(0.7)	0(0)	220(31.5)	30(31.6)	32(31.7)	223(12.1)	31(13.0)	31(14.6)
Margins									
Smooth/ Ill-defined	992(86.6)	108(75.5)	85(78.9)	477(68.3)	60(63.2)	62(61.4)	1469(79.7)	168(70.6)	147(69.0)
Lobulated/ irregular	153(13.3)	35(37.5)	27(24.1)	210(30.1)	30(31.5)	32(31.7)	363(19.7)	65(27.3)	59(27.7)
Extra-thyroid extension	1(0.1)	0(0)	0(0)	11(1.6)	5(5.3)	7(6.9)	12(0.6)	5(2.1)	7(3.3)
Echogenic foci									
None/large comet-tail artifacts	991(86.5)	122(85.3)	93(83.0)	92(13.2)	16(16.8)	19(18.8)	1083(58.7)	138(58.0)	112(52.6)
Macrocalcifications	133(11.6)	16(11.2)	10(8.9)	17(2.4)	5(5.3)	3(3.0)	150(8.1)	21(8.8)	13(6.1)
Peripheral calcifications	22(1.9)	6(4.2)	6(5.4)	3(0.4)	0(0)	1(1.0)	25(1.4)	6(2.5)	7(3.3)
Punctate echogenic foci	22(1.9)	7(4.9)	5(4.5)	601(86.1)	76(80.0)	78(77.2)	623(33.8)	83(34.9)	83(39.0)

*Test set A and test set B referred to the internal test set and external test set. Data in parentheses are percentages.

### DL Performance Compared With Radiologists

The performance of DL was better compared to the radiologists in three tasks. In the internal test set, the AUROC of the best algorithm in differentiation of thyroid nodules was 0.936 (95%CI 0.898-0.973) in TR4, 0.915 (95%CI 0.857-0.973) in TR5 and 0.892 (95%CI 0.850-0.933) in TR 4&5 respectively, which overwhelmingly exceeded the radiologists respectively (P < 0.001). In the external test set, the AUROC of the optimal algorithm was 0.904 (95%CI 0.833-0.951) in TR4, 0.845 (95%CI 0.759-0.909) in TR5 and 0.829 (95%CI 0.772-0.877) in TR 4&5 respectively, which again was better than the radiologists (P < 0.001).

Evaluation of the performance on differentiation of malignant from benign thyroid nodules in TR4, TR 5 and TR 4&5 were recorded in **Tables 3**–**5**, respectively. ResNet-50 performed best in the certain classification in both TR4 and TR5 dataset. Meanwhile, performance in two datasets was also excellent with a stable repeatability, of which the kappa value was all over 0.50.

**Table 3:** Performance of deep learning containing three CNNs compared with the radiologists in differentiating benign and malignant thyroid nodules classified into ACR TI-RADS category 4.

	ResNet-50	Inception-	Desnet-121	Radiologists	*P*
		Resnet-v2			value
Internal dataset (n=143)					
Accuracy	0.874 (0.810-0.919)	0.846 (0.778-0.896)	0.846 (0.778-0.896)	0.734 (0.656-0.800)	0.010
Sensitivity	0.836 (0.727-0.909)	0.918 (0.824-0.966)	0.863 (0.758-0.929)	0.684 (0.564-0.786)	0.004
Specificity	0.914 (0.816-0.965)	0.771 (0.653-0.860)	0.871 (0.765-0.936)	0.786 (0.668-0.871)	0.066
PPV	0.910 (0.809-0.963)	0.807 (0.703-0.883)	0.875 (0.771-0.938)	0.769 (0.645-0.861)	0.115
NPV	0.842 (0.736-0.912)	0.900 (0.788-0.959)	0.859 (0.752-0.927)	0.705 (0.590-0.800)	0.024
Kappa value	0.749	0.691	0.693	0.470	
F_1_	0.846	0.775	0.846	0.649	
AUROC	0.936 (0.898-0.973)	0.902 (0.853-0.952)	0.911 (0.865-0.958)	0.735 (0.652-0.819)	
External dataset (n=112)					
Accuracy	0.830 (0.749- 0.890)	0.821 (0.739-0.882)	0.795 (0.710-0.860)	0.741 (0.653 -0.814)	0.033
Sensitivity	0.829 (0.657-0.928)	0.657 (0.477-0.803)	0.800 (0.625-0.909)	0.686 (0.506-0.826)	0.108
Specificity	0.831 (0.725-0.904)	0.896 (0.800-0.951)	0.792 (0.682-0.873)	0.766 (0.653-0.852)	0.101
PPV	0.690 (0.528-0.819)	0.742 (0.551-0.875)	0.636 (0.477-0.772)	0.571 (0.410-0.719)	0.037
NPV	0.914 (0.816-0.965)	0.852 (0.752-0.918)	0.897 (0.793-0.954)	0.843 (0.732-0.915)	0.226
Kappa value	0.626	0.571	0.553	0.429	
F_1_	0.812	0.785	0.775	0.713	
AUROC	0.904 (0.833-0.951)	0.845 (0.765-0.907)	0.842 (0.761-0.904)	0.726 (0.634-0.806)	

**Table 4:** Performance of deep learning containing three CNNs compared with the radiologists in differentiating benign and malignant thyroid nodules classified into ACR TI-RADS category 5.

	ResNet-50	Inception-	Desnet-121	Radiologists	*P*
		Resnet-v2			value
Internal dataset (n=95)					
Accuracy	0.863 (0.780-0.918)	0.811 (0.720-0.877)	0.832 (0.744-0.894)	0.695 (0.596-0.778)	0.022
Sensitivity	0.841 (0.723-0.917)	0.841 (0.723-0.917)	0.952 (0.858-0.988)	0.635 (0.504-0.750)	<0.001
Specificity	0.906 (0.738-0.975)	0.750 (0.562-0.879)	0.594 (0.408-0.758)	0.813 (0.630-0.921)	0.026
PPV	0.946 (0.842-0.986)	0.869 (0.752-0.938)	0.822 (0.711-0.898)	0.870 (0.730-0.946)	0.055
NPV	0.744 (0.576-0.864)	0.706 (0.523-0.843)	0.864 (0.640-0.964)	0.531 (0.384-0.672)	0.026
Kappa value	0.709	0.592	0.582	0.396	
F_1_	0.854	0.791	0.793	0.688	
AUROC	0.915 (0.857-0.973)	0.838 (0.756-0.919)	0.906 (0.846-0.966)	0.724 (0.617-0.831)	
External dataset (n=101)					
Accuracy	0.822 (0.735-0.885)	0.713 (0.618 to 0.792)	0.802 (0.713-0.869)	0.703 (0.607-0.784)	0.080
Sensitivity	0.846 (0.731-0.920)	0.615 (0.486-0.731)	0.754 (0.629-0.849)	0.677 (0.548-0.785)	0.211
Specificity	0.778 (0.604-0.893)	0.889 (0.730-0.964)	0.889 (0.730-0.964)	0.750 (0.575-0.873)	0.128
PPV	0.873 (0.760-0.940)	0.909 (0.774-0.970)	0.925 (0.809-0.976)	0.830 (0.697-0.915)	0.132
NPV	0.737 (0.566-0.860)	0.561 (0.424-0.690)	0.667 (0.515-0.792)	0.563 (0.413-0.702)	0.203
Kappa value	0.616	0.446	0.598	0.397	
F_1_	0.808	0.711	0.796	0.694	
AUROC	0.845 (0.759-0.909)	0.770 (0.676-0.848)	0.842 (0.756-0.907)	0.713 (0.615-0.799)	

**Table 5:** Performance of deep learning containing three CNNs compared with the radiologists in differentiating benign and malignant thyroid nodules classified into ACR TI-RADS category 4 and 5.

	ResNet-50	Inception-	Desnet-121	Radiologists	*P*
		Resnet-v2			value
Internal dataset (n=238)					
Accuracy	0.832 (0.779-0.874)	0.811 (0.756-0.856)	0.824 (0.770-0.867)	0.718 (0.658-0.772)	0.007
Sensitivity	0.882 (0.813-0.929)	0.794 (0.715-0.857)	0.824 (0.747-0.882)	0.662 (0.711-0.898)	<0.001
Specificity	0.745 (0.647-0.824)	0.833 (0.744-0.897)	0.843 (0.755-0.905)	0.794 (0.700-0.865)	0.227
PPV	0.822 (0.748-0.878)	0.864 (0.788-0.916)	0.875 (0.802-0.925)	0.811 (0.723-0.877)	0.429
NPV	0.826 (0.730-0.894)	0.752 (0.660-0.826)	0.782 (0.691-0.853)	0.638 (0.547-0.720)	0.009
Kappa value	0.635	0.619	0.660	0.442	
F_1_	0.852	0.784	0.836	0.668	
AUROC	0.879 (0.835-0.922)	0.883 (0.841-0.926)	0.892 (0.850-0.933)	0.728 (0.663-0.793)	
External dataset (n=213)					
Accuracy	0.784 (0.724-0.834)	0.770 (0.709-0.822)	0.761 (0.699-0.813)	0.723 (0.659-0.779)	0.009
Sensitivity	0.790 (0.695-0.862)	0.860 (0.773-0.919)	0.710 (0.609-0.794)	0.680 (0.578-0.768)	0.023
Specificity	0.779 (0.689-0.849)	0.690 (0.595-0.772)	0.805 (0.718-0.871)	0.761 (0.670-0.834)	0.530
PPV	0.760 (0.664-0.836)	0.711 (0.620-0.788)	0.763 (0.662-0.843)	0.716 (0.613-0.801)	0.055
NPV	0.807 (0.718-0.874)	0.848 (0.754-0.911)	0.758 (0.670-0.830)	0.729 (0.638-0.805)	0.071
Kappa value	0.567	0.544	0.517	0.442	
F_1_	0.784	0.770	0.758	0.722	
AUROC	0.829 (0.772-0.877)	0.807 (0.748-0.858)	0.793 (0.733-0.845)	0.721 (0.655-0.780)	

### Heatmaps Generated by CAM

**Figure 2:**
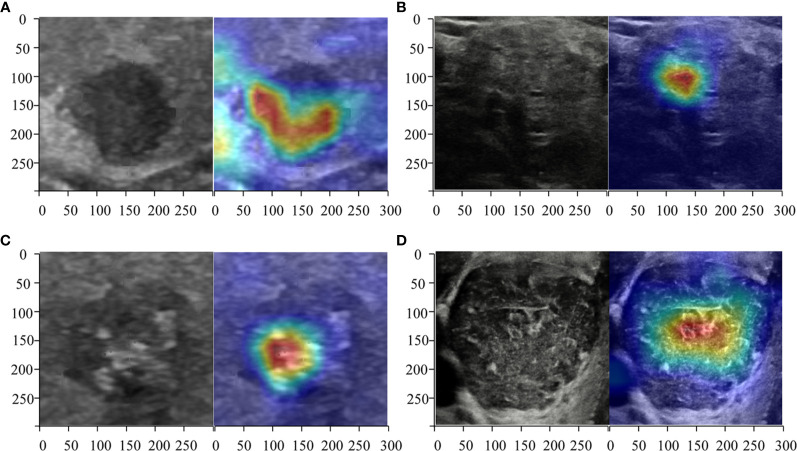
Heatmaps of the region of interest (ROI) of the thyroid nodules using class activation mapping (CAM). The red color showed the prediction regions the CNNs focused which estimated to be determined as the thyroid cancer. Three radiologists and DL correctly predicted a malignant **(A)** thyroid nodule diagnosed as micro papillary carcinoma TR4 and a benign **(B)** one diagnosed as non-toxic nodular goiter of TR4. ResNet50, Desnet121, and the radiologists deemed a malignant nodule **(C)** diagnosed as papillary carcinoma of TR5 as malignance but a DL algorithm named Inception-ResNet version 2 judged it as benign. All CNNs correctly predicted a benign **(D)** thyroid nodule diagnosed as Hashimoto’s thyroiditis of TR5 but the radiologists all predicted wrongly.

Heatmaps were generated to present the recognition pattern of the deep learning model as demonstrated in **Figure 2**. The greatest predictive regions of the tumor CNNs concentrated were shown as red and yellow; whereas the areas green and blue regions were of less predictive significance. This shows that the DL algorithms focuses on the most predictive image features of thyroid nodules malignance risk.

## Discussion

In this study, we combined ACR TI-RADS with DL by training three commonly used deep learning algorithms to discriminate between benign and malignant in TR4 and TR5 thyroid nodules with available pathology. As shown in **Figure 3**, no matter which type of TI-RADS was used for the classification competition, DL algorithms performed better than radiologists. The accuracy in all models was higher in TR4 and TR5 for test set A and test set B, which was parallel to the performance of the radiologists. However, in the case of mixing different feature sets containing TR4 and TR5, DL still had good performance but slightly weaker than the two separated sets, which might be related to more complex tasks.

Patients with suspected thyroid nodules, nodular goiter, nodules accidentally discovered by radiological examination such as computed tomography (CT), magnetic resonance imaging (MRI), or 18F-flurodeoxyglucose positron emission computed tomography (FDP18-PET) scan showing thyroid uptake should undergo diagnostic thyroid ultrasound examination as recommended by ATA Guidelines 2015 (26). The benign and malignant ultrasound results of nodules will determine whether FNA and follow-up are to be carried out (27), and the choice of treatment methods will be influenced by ultrasound opinions and cervical lymph node conditions (28). In ultrasound diagnosis, malignant nodules have various manifestations and particularly those with atypical appearances and fuzzy boundaries lead to diagnostic difficulties (29, 30). Radiologists frequently disagree over the interpretation of these malignant tumors. DL may provide assistance for radiologists with good accuracy and consistency.

The performance of DL is often better than that of radiologists and even machine learning, in the diagnosis of thyroid nodules. Xia and colleagues (31) achieved an accuracy of 87.7% in differentiating malignant and benign nodules by constructing extreme machine learning based on collected features obtained from 203 ultrasound images of 187 patients with thyroid cancer. Li and colleagues (19) got an accuracy of 89.8% (95% CI 86.8–92.3) in internal validation set with the DCNN model versus 78.8% with the radiologists and 85.7% (95% CI 79.2–90.8) versus 72.7% (65.0–79.6%) in external validation set. Machine learning gives opinions by extracting computational features and calculating statistically significant finite features and modeling. The modeling process of machine learning requires the segmentation of images to be more accurate, while the commonly manual work is difficult to control. Limited quantities of features and smaller sample size also resulted in inferior performance and narrow application range.

Moreover, the DL result in thyroid nodules of all TR categories was not that impressive because it contained some tasks that even radiological beginners can do such as recognizing and selecting the TR1 nodules and labelling them as benign (5). Limiting the work to differentiation between subtype TR4 and TR5 is difficult for radiologists because they had similar visible features (20). As recent studies have reported, DL had achieved great success on the classification on thyroid cancer (32), when all types of thyroid nodules were included. In these studies, pathological results of some nodules were not available (19), while in our study all the nodules correlated with surgical pathology. Limitations of the TR categories on ultrasound images avoid heterogeneity of the dataset to a degree. In specific classification, our study revealed that a precise set of certain categories contributed to the higher accuracy compared with former studies (19, 32).

The result of this study may potentially be of clinical value. TI-RADS is already widely applied worldwide and combining the TI-RADS and DL provides more accurate results and should be easily accepted clinically. Previous studies had reported that interobserver agreement in the lexicon was also substantial thus the pre-classification was easily performed and credible wherever used (33). Application of the DL based on ACR TI-RADS will supply useful suggestions when there is doubt over the diagnosis and will support services where medical resources were unbalanced.

Our study also had limitations. First, this was a retrospective study with limited categories of data. The performance of our DL system is expected to increase by including more data and expanding several sets from other hospitals. And exclusion of TR3 thyroid nodules decrease clinical application to some extent. Second, ultrasound systems of different manufactures and heterogeneity of operators may give rise to the variability in the training process. The inter-reader reliability of nodule extraction was not assessed. Third, the images reviewed were static in this study that features from multi-sections were not considered.

To be summarized, the study demonstrated that DL based on ACR TI-RADS could improve the differentiation of malignant from benign thyroid nodules with great clinical application potential. With a stable repeatability, DL algorithms showed better performance than radiologists for TNs of TR4 and TR5 categories, which are the most difficult categories for diagnosis in clinical practice. Prospective studies with long-term follow-up will be needed to examine the utility of the system and assess its effectiveness in routine clinical practice.

## Data Availability Statement

The raw data supporting the conclusions of this article will be made available by the authors, without undue reservation.

## Ethics Statement

The studies involving human participants were reviewed and approved by Ethics Committee of Tongji Medical College of Huazhong University of Science and Technology. Written informed consent for participation was not required for this study in accordance with the national legislation and the institutional requirements.

## Author Contributions

Guarantors of integrity of entire study: G-GW, W-ZL, RY, X-WC, and BZ. Literature research: G-GW, W-ZL, RY, J-YW, X-WC, and BZ. Study concepts/study design: all authors. Contributed to acquisition of data: G-GW, W-ZL, RY,Y-JY, and BZ. Clinical studies: G-GW, RY, J-WX, Y-JY, R-XC, X-WC, and BZ. Contributed reagents/materials/analysis tools: G-GW, W-ZL. Manuscript drafting or manuscript revision: all authors. Statistical analysis: G-GW, W-ZL, RY, J-WX, Y-JY, R-XC, X-WC, and BZ. All authors contributed to the article and approved the submitted version.

## Funding

This work was supported Natural Science Foundation of Hubei Province (2019CFB286), Natural Science Foundation of Hunan Province (2017JJ2394), Corps Science and Technology Key Project (2019DB012).

## Conflict of Interest

The authors declare that the research was conducted in the absence of any commercial or financial relationships that could be construed as a potential conflict of interest.
